# The prevalence and severity of upper gastrointestinal complications among patients with chronic diseases: a cross-sectional study from Palestine

**DOI:** 10.1186/s12876-024-03267-y

**Published:** 2024-05-21

**Authors:** Rowa Al Ramahi, Deema Tumeh

**Affiliations:** Department of Pharmacy, Faculty of Medicine and Health Sciences, An-Najah National University, P.O.Box 7, Nablus, Palestine

**Keywords:** Chronic disease, Upper gastrointestinal side effects, Stomach irritation, Dyspepsia, Palestine

## Abstract

**Background:**

Many old people have at least one chronic disease. As a result, multiple drugs should be used. Gastrointestinal complications may occur because of the harmful effects of these chronic drugs on the stomach. The study aimed to assess the prevalence of upper gastrointestinal complications in patients taking chronic medications, the severity of these symptoms, and whether they take any gastro-protective drugs or not.

**Methodology:**

This was a cross-sectional study through face-to-face questionnaires from internal outpatient clinics at a specialized hospital. Patients with chronic diseases who were taking at least one chronic medication were included in the study. Data Collection Form was used to gather information. The Short-Form Leeds Dyspepsia Questionnaire (SF-LDQ) was used to evaluate the severity of the upper gastrointestinal symptoms. Statistical analysis was performed using the Statistical Package for Social Sciences (SPSS) version 21.

**Results:**

A total of 400 patients with chronic diseases and using multiple medications were included. Among them, 53.8% were females and 56% were married, 58.5% were unemployed, 70% were not smokers, the mean age was 54.7 ± 17.5 years. The most common comorbid diseases among the patients were diabetes, hypertension, and arthritis, with percentages of 44.3%, 38%, and 27.3%, respectively. The mean number of chronic medications used was 3.36 ± 1.6 with a range of 1 to 9. The most commonly used was aspirin with a percentage of 50%, followed by atorvastatin, bisoprolol, and insulin with percentages of 29.5%, 25%, and 20.3%, respectively. Among the 400 participants, 362 (90.5%) suffered from upper gastrointestinal side effects like indigestion (65.8%), heartburn (78.3%), nausea (48.8%), and regurgitation (52.0%). Based on SF-LDQ scoring, of the 400 respondents, 235(58.8%), 109(27.3%) and 18(4.5%) suffered from mild, moderate and severe dyspepsia, respectively. A high percentage 325 (81.3%) of participants were prescribed gastro-protective medications. Proton pump inhibitors were the most prescribed group in 209 (52.3%) patients. Dyspepsia was significantly associated with older age (*p*-value = 0.001), being educated (*p*-value = 0.031), not being single (*p*-value < 0.001), having health insurance (*p*-value = 0.021), being a smoker (*p*-value = 0.003), and using ≥ 5 medications (*p*-value < 0.001).

**Conclusion:**

Upper gastrointestinal complications among patients with chronic diseases were very common. Fortunately, the symptoms were mild in most cases. The risk increased with age and using a higher number of medications. It is important to review patients’ medications and avoid overuse of them, in addition to use gastro-protective agents when needed.

## Introduction

In recent years, the number of chronic diseases has increased as a consequence of the increased number of elderly people and life style [1]. Older population is more susceptible to multiple drug therapy [2]. The gastrointestinal (GI) tract, especially the stomach, has protective defense mechanisms that can withstand the harmful effects, but chronic exposure is the problem. The mucosal lining of the stomach, the generation of prostaglandin and the repair effect are the main protective mechanisms. Some drugs like aspirin and Non-Steroidal Anti-inflammatory Drugs (NSAIDs) can inhibit the synthesis of prostaglandins by inhibiting the cyclooxygenase enzyme and so the mucosal defense. In addition, chronic use of any drug may produce cytotoxic compounds that irritate the mucosal lining of the stomach and cause dyspepsia and ulcers [3].

Chronic diseases as cardiovascular diseases, stroke, cancer, arthritis, and type 2 diabetes are very common among elderly [4]. Many medications as cardiovascular drugs, analgesics, hematopoietic drugs, gastrointestinal drugs and endocrine drugs are widely used on chronic bases [5].

According to the Health Annual Report for 2020, of all the deaths in Palestine in 2020, 24.7% were due to cardiovascular diseases, 14.6% due to diabetes mellitus, 10.7% due to cerebrovascular diseases, and 1.6% due to chronic obstructive pulmonary disease [6].

Dyspepsia and heartburn are considered the main consequences of chronic drug use. Studies have shown that upper GI symptoms including dyspepsia occurred in patients who were taking NSAIDs at a relative risk of about 1.5 to 2 compared with that in patients who do not use them [7]. Heartburn recognized as a retrosternal burning sensation had a prevalence of 12.9% among the elderly using NSAIDs [8]. Nausea is also considered a main side effect of many drugs like analgesics, cardiovascular medications, digoxin, antiarrhythmic drugs, antihypertensive, beta blockers, calcium-channel antagonists, hormonal preparations/therapies, oral antidiabetics and many more. It is defined as a precursor sensation of vomiting, a desire to expel stomach contents [9]. These upper GI symptoms might not be dangerous, but they are bothersome and may affect daily activities and adherence to medications [10]. In a previous study from our country, a very high percentage of university students admitted using NSAIDs although most of them were aware of the side effects on the gastrointestinal system, kidneys and cardiovascular system [11].

Using multiple drugs is common in the elderly population. In a previous study, 17.3% of the elderly used five or more drugs, 11.7% used four drugs, 18.5% used three drugs. Furthermore, there was a significant correlation between the use of multiple drugs and the appearance of different side effects, indicating that the doctor should use the fewest drugs possible [5].

The study aimed to assess the prevalence of upper gastrointestinal complications in patients taking chronic drugs, the severity of these symptoms, and whether they take any gastro-protective drugs or not. To the best of our knowledge, this study is the first of its type in Palestine. So, the findings will give base line data about GI symptoms with chronic medications and open the eyes of prescribers to consider this and treat it if present which may help doctors and policy-makers in their efforts to limit drug-related problems.

## Methodology

### Study design

This study was a cross-sectional questionnaire-based study. A face-to-face interview was used to measure the prevalence of upper gastrointestinal complications in patients using chronic medications, the type of these complications, and which treatments were used in these cases. It was conducted between October 2021 to May 2022.

### Study setting

Palestine consists of two geographically separated zones – the Gaza Strip and the West Bank – with a total population of about five million inhabitants. Nearly 61.5% live in the West Bank and 38.5% live in the Gaza strip. This study was conducted in Jenin city in the West Bank and included patients visiting outpatient internal clinics in Ibn Sina hospital. The population of this study was patients of both genders with any chronic disease from outpatient clinics.

**Table 1:** Socio-demographic and clinical characteristics of the study sample

Socio-demographic Variables		Frequency (%) *N* = 400
**Gender**	Male Female	185(46.2) 215(53.8)
**Age**	< 40 40–64 65 or more	86(21.5%) 190(47.5%) 124(31%)
**Educational status**	Primary Precursory Secondary Undergraduate Postgraduate	90(22.5) 66(16.5) 78(19.5) 145(36.2) 21(5.3)
**Social status**	Single Married Divorced Widower	38(9.5) 224(56.0) 41(10.3) 97(24.2)
**Income/month (Jordanian Dinar)**	less than 400 400–600 601–800 801–1000 more than 1000	22(5.5) 71(17.8) 136(34.0) 110(27.5) 61(15.2)
**Employment status**	Employed Unemployed	165(41.4) 234(58.6)
**Locality**	City Village Camp	139(34.8) 186(46.5) 75(18.7)
**Health insurance**	Don’t have insurance Have insurance	126(31.5) 274(68.5)
**Smoking**	Non smoker Smoker	283(70.8) 117(29.2)

**Table 2:** Frequency and percentage of gastrointestinal side effects

Symptoms	Percentage	Response category%				
		Not at all	Less than monthly	Between monthly and weekly	Between weekly and daily	More than daily
Indigestion frequency	263(65.8)	137(34.3)	98(24.5)	104(26.0)	51(12.8)	10(2.5)
Heartburn frequency	313(78.3)	87(21.8)	95(23.5)	134(33.5)	65(16.3)	19(4.8)
Regurgitation frequency	208(52.0)	192(48.0)	124(31.1)	43(10.8)	28(7.0)	13(3.3)
Nausea frequency	195(48.8)	205(51.3)	98(24.5)	65(16.3)	24(6.0)	8(2.0)
Indigestion severity	-	230(57.5)	112(28)	42(10.5)	15(3.8)	1(0.25)
Heartburn severity	-	177(44.3)	1(0.3)	127(31.8)	72(18.1)	23(5.8)
Regurgitation severity	-	298(74.6)	68(17.0)	21(5.3)	12(3.0)	1(0.3)
Nausea severity	-	280(70.0)	80(20.0)	31(7.8)	7(1.8)	2(0.5)

**Table 3:** *Association between dyspepsia and socio-demographic characteristics of the patients estimated by univariate analysis* (*n = 400)*

Variables		No dyspepsia (%)	Mild dyspepsia (%)	Moderate dyspepsia (%)	Severe dyspepsia (%)	*P* value*
Age	< 40	16(18.6)	51(59.3)	18(20.9)	1(1.2)	**0.001**
	40–64	16(8.4)	117(61.6)	44(23.2)	13(6.8)	
	65 or more	6(4.8)	67(54.0)	47(37.9)	4(3.2)	
Gender	Male	16(8.6)	104(56.2)	56(30.3)	9(4.9)	0.595
	Female	22(10.2)	131(60.9)	53(24.7)	9(4.2)	
Education	Primary	3(3.3)	50(55.6)	33(36.7)	4(4.4)	**0.031**
	Precursory	5(7.6)	40(60.6)	20(30.3)	1(1.5)	
	Secondary	11(14.1)	55(70.5)	10(12.8)	2(2.6)	
	Undergraduate	17(11.7)	80(55.2)	39(26.9)	9(6.2)	
	Postgraduate	2(9.5)	10(47.6)	7(33.3)	2(9.5)	
Social status	Single	10(26.3)	21(55.3)	7(18.4)	0(0.0)	**< 0.001**
	Married	19(8.5)	136(60.7)	62(27.7)	7(3.1)	
	Divorced	5(12.2)	27(65.9)	5(12.2)	4(9.8)	
	Widower	4(4.1)	51(52.6)	35(36.1)	7(7.2)	
Living place	City	16(11.5)	82(59.0)	35(25.2)	6(4.3)	0.849
	Village	16(8.6)	108(58.1)	55(29.6)	7(3.8)	
	Camp	6(8.0)	45(60.0)	19(25.3)	5(6.7)	
Health insurance	Yes	19(6.9)	164(59.9)	75(27.4)	16(5.8)	**0.021**
	No	19(15.1)	71(56.3)	34(27.0)	2(1.6)	
Monthly income	< 400	2(9.1)	14(63.6)	5(22.7)	1(4.5)	0.395
	600 − 400	3(4.2)	40(56.3)	25(35.2)	3(4.2)	
	800 − 601	15(11.0)	76(55.9)	39(28.7)	6(4.4)	
	801–1000	7(6.4)	69(62.7)	28(25.5)	6(5.5)	
	> 1000	11(18.0)	36(59.0)	12(19.7)	2(3.3)	
Employment	Yes	16(9.7)	91(55.2)	47(28.5)	11(6.7)	0.373
	No	22(9.4)	144(61.5)	62(26.1)	7(3.0)	
Smoking	Yes	8(6.8)	56(47.9)	45(38.5)	8(6.8)	**0.003**
	No	30(10.6)	179(63.3)	64(22.6)	10(3.5)	
Number of medications	< 5	38(11.8)	208(64.6)	73(22.7)	3(0.9)	**< 0.001**
	5 or more	0(0.0)	27(34.6)	36(46.2)	15(19.2)	

*Chi-Square test

### Ethical approval

All aspects of the study protocol, including access to and use of patients’ clinical information, were authorized by the Institutional Review Boards (IRBs) of our university (Reference number: Mas. Oct. 2021/35) and the administration of the hospital before the initiation of the study. In addition, a written informed consent was obtained from each patient.

### Sample size calculation and sampling procedure

An automated software program, Raosoft sample size calculator (http://www.raosoft.com/samplesize.html) was used to calculate the required sample size for this study. The sample size needed was calculated using 50% as a response distribution to achieve a confidence level of 95% and a margin of error of 5%. The estimated sample size was 385 patients, so we included 400 patients. Convenience sampling was used to recruit participants.

### Inclusion and exclusion criteria

The inclusion criteria were as follows: males and females, confirmed diagnosis of chronic diseases and using one or more chronic medications. The exclusion criteria were as follows: patients who did not have chronic diseases or did not use chronic medications for them, patients with current cancer treatment and pregnant women.

### Data collection

Data collection was standardized by using a Data Collection Form to gather information from questionnaires and patients’ files which was developed based on previous studies [10, 12–15]. The data included sex, age, the type of chronic disease, names and numbers of medications used, gastrointestinal complications, the severity of this complication, if gastro-protective treatment is taken, the type of gastro-protective treatment. To evaluate the severity, The Arabic version of Short-Form Leeds Dyspepsia Questionnaire (SF-LDQ) was used which was validated in a previous study [15], the validity and reliability of the English version was tested in a previous study where the Pearson coefficient for test-retest reliability was 0.93 and it had a sensitivity of 77% and a specificity of 75%, so it was considered as a reliable and a valid self-completed outcome measure for quantifying the frequency and severity of dyspepsia symptoms [12]. The validation of the Arabic version showed that using clinician’s diagnosis for concurrent validity led to 69.49% sensitivity and 83.24% specificity of SF-LDQ. This indicates that the Arabic validated version of SF-LDQ can be used to screen for dyspepsia in the Arabic speaking population [15] It includes 9 validated questions, the questionnaire score lies between (0–32), as zero represents no dyspepsia, a score between (1–8) represents mild dyspepsia, a score of (9–15) represents moderate dyspepsia and a sore of (15–32) represents severe dyspepsia [10].

A pilot study (30 participants) was conducted to test the tool, ensure the availability of the required data, estimate the time, and modify the data collection form, as appropriate. The patients participating in the pilot study were not included in the final analysis. The average time to complete the survey by the patient was around 10 min. Data collection was done by a postgraduate student in clinical pharmacy, she had required directions and training before starting data collection.

### Statistical analysis

The Statistical Package for Social Sciences program version 21 (SPSS) was used to analyze the data. Data was expressed as mean ± SD for continuous variables and as frequencies (percentages) for categorical variables. A univariate analysis was conducted to determine the relationship between the prevalence of dyspepsia and the patients’ characteristics; Chi-square test was used to test for significant associations between categorical variables. The significance level was set at a *p*-value of 0.05.

### Results

Patients who were approached were 450, among them 400 patients accepted to participate giving a response rate of 88.9%. These patients were diagnosed with chronic diseases and were using one or more chronic medications. Table 1 shows that more than half of the patients were females (53.8%), mean age was 54.7 ± 17.5 years old (Range: 14–85 years), 56.0% of them were married, 36.3% were undergraduates, 58.5% were unemployed, the monthly income of the family was between 601 and 800 Jordanian Dinar for 34% of them, among the sampled patients, 68.5% had health insurance, 46.5% of them were living in a village and 70.8% of the participants were nonsmokers.

This study focused on patients with chronic diseases and used multiple medications. The most common chronic disease among the patients was diabetes mellitus in 177 (44.3%), followed by hypertension in 152 (38%), then arthritis in 109 (27.3%), dyslipidemia in 77 (19.3%) and ischemic heart disease in 34 (8.5%) patients. The mean number of chronic medications used was 3.36 ± 1.6 with a range of 1 to 9. Many patients used 3 medications (123, 30.8%), followed by 2 medications (93, 23.3%), then 4 medications (73, 18.3%), then 6 medications (31, 7.8%). The most commonly used drugs by the patients in our sample were aspirin with a percent of 50%, followed by atorvastatin, bisoprolol, insulin and metformin with a percent of (29.5%, 25.0%, 20.3% and 14.8%) respectively.

Among the 400 participants who were enrolled in the study, 362 (90.5%) had some degree of dyspepsia (indigestion, regurgitation, heartburn, or nausea). Table 2 shows the frequency and severity of these gastrointestinal side effects; 263 (65.8%) of the patients had indigestion, which occurred mainly once weekly (26%). On the other hand, 313 (78.3%) of the patients had heartburn that occurred once weekly with a percent of 33.5%. Regarding regurgitation, 208 (52.0%) of the patients had it once monthly with a percent of 31.1%. Also, 195(48.8%) of the patients had nausea, which occurred mainly once monthly (24.5%).

When we tried to find any possible association between using certain medications and dyspepsia, most medications did not show statistically significant associations except for metformin as all users 59 (100%) reported dyspepsia while this was in 303 (88.9%) of non-users (*p*-value = 0.007) and for atorvastatin as 115 (97.5%) of users reported dyspepsia versus 247(87.6%) of not users (*p*-value = 0.002).

Based on the Short-Form Leeds Dyspepsia Questionnaire (SF-LDQ) scoring, of the 400 respondents, 235(58.8%), 109(27.3%) and 18(4.5%) suffered from mild, moderate and severe dyspepsia, respectively.

As shown in Table 3, there were no significant associations between dyspepsia and gender, living place, monthly income, or working status (*p*-values > 0.05). On the other hand, dyspepsia was significantly associated with older age (*p*-value = 0.001), being educated (*p*-value = 0.031), not being single (*p*-value < 0.001), having health insurance (*p*-value = 0.021), being a smoker (*p*-value = 0.003), and using ≥ 5 medications (*p*-value < 0.001). A very high percentage 325 (81.3%) of the patients with chronic diseases used a drug to protect the stomach which was prescribed by their doctors, most of them 209 (52.3%) were prescribed proton pump inhibitors, 10% used histamine (H2) receptor blocker and 13.3% used sodium alginate/potassium bicarbonate, 3.5% used bismuth, 1% used aluminum hydroxide/magnesium hydroxide, 3.3% used famotidine/magnesium hydroxide/calcium bicarbonate. All the previous medications were used to treat acid secretion. Furthermore, 16% used mebeverine as antispasmodic, 16% used domperidone as anti- sickness, 1.8% used metoclopramide for nausea and vomiting, 4.5% used clordiazepoxide/clinidium bromide to decrease hypersecretion and hypermobility in the gastrointestinal tract, 6.5% used sulpiride for irritable bowel syndrome and duodenal ulcers.

### Discussion

This study was one of the first in Palestine to examine whether there is any relationship between the use of chronic medications and the patients’ gastrointestinal side effects, using a questionnaire to determine the prevalence of patients with GI complications and their severity, and to determine the percentage of patients who used gastro-protective drugs and medications.

Most of the participants were married, middle-aged, educated, unemployed, living in a village, non-smokers, and had health insurance. Furthermore, the majority of them had multiple chronic diseases, with diabetes being the most common, followed by hypertension and arthritis, which is similar to a previous study conducted in Palestine that revealed that living in the West Bank was a risk factor for chronic diseases such as diabetes, hypertension, and CVD but not for cancer, and that being female, married, and being over 40 were risk factors for chronic diseases [16].

This study found that most patients used 2–4 medications daily; the mean number was 3.36 ± 1.6. Using many medications was seen in other studies especially those among old people [17]. Half of our patients used aspirin, which corresponds to a study performed in Palestine on 1192 participants; 48% were taking aspirin as one of their medications. Furthermore, a high percent of atorvastatin, bisoprolol, and insulin were used due to a widespread percent of cardiovascular diseases and endocrine diseases (mainly diabetes) among Palestinians [18].

More than 90% of our participants reported some degree of dyspepsia. This is an alarming percentage that requires special attention when multiple medications are prescribed especially for elderly. This differs from the prevalence of neighboring Arabian countries, as reported in Jordan by Frasakh et al. (60.1%) [13] and as reported in UAE by Jaber et al. (44%) [14]. It is important to note that the Jordanian study was among general population and the Emirati study was among pre-clinical medical students. Different populations are expected to have different prevalence. High prevalence of dyspepsia in this study was similar to results from Saudi Arabia, dyspepsia percentage was 92.4% as reported by Alwhaibi et al. [10].

The good news that 58% of our participants had mild dyspepsia (1–8) score which resembles the findings of Saudi Arabian study as 41% had mild dyspepsia [10]. These mild symptoms might be explained by the wide use of gastro-protective medications which may help in decreasing the severity of symptoms.

Older age had a significant association with dyspepsia (*p*-value < 0.05), this may be due to high probability to H pylori infection or the use of higher number of medications [19, 20]. Being a smoker also had a significant association [21]. However, high education level and being married were considered protective factors as mentioned in previous studies [22]. Having health insurance was significantly related to dyspepsia, most likely patients with chronic diseases using many medications will seek to have a medical insurance to cover the expenses of their diseases [23, 24].

Using five or more medications was significantly associated with dyspepsia. This could be due to stomach irritation caused by the use of multiple medications. The harmful effects of chronic medications on the stomach have been emphasized in previous studies which showed that continuous exposure to multiple medications like aspirin and NSAIDs can inhibit prostaglandin synthesis, which plays an important role in all gastrointestinal defense mechanisms [3]. In addition, chronic medication use leads to accumulation of Reactive Oxygen Species (ROS) in the GIT and an inability to scavenge them by melatonin [25]. The prescribers are recommended to use the least possible number of medications and formulations that may have less GI side effects as enteric-coated tablets.

In our population, the most common and bothersome gastrointestinal side effect was heartburn; it affected the daily activities for a wide range of people. The harmful effect on the gastrointestinal tract may result from a drug’s mode of action, through direct injury, by changing the mucosal integrity, or as a result of changes in colonic microbiota [26]. Gastroprotective medications can help in controlling symptoms. A very high percentage of patients were using one of them especially proton pump inhibitors that were prescribed for 52.3%, this high rate requires further studies to review the rational of this use and if their extra cost and possible side effects are justified.

In summary, this study found that upper GI symptoms are very common among patients with chronic diseases especially if they are old and use a high number of medications, so prescribers and pharmacists need to ask about these side effects and try to minimize them by using the lowest number and doses of medications, using enteric-coated formulations when possible, using gastroprotective medications when needed. In addition to suitable counseling regarding lifestyle and suitable diet.

Regarding strength of this study, to the best of our knowledge, this study was the first to investigate the effect of chronic drug use on the gastrointestinal tract in Palestine, furthermore it investigated the main gastro protective drugs used in these patients. The limitations include the design as it was a cross-sectional study, so as a result, it is difficult to prove causal relationships between the questions and their associated factors. It did not explore other confounding factors like work, adherence, and food. Recall bias; as the sample was mainly older people, so they may face some difficulty in remembering information. In addition, the results were from one hospital only, so the results may not be representative to the entire Palestinian population.

### Conclusions

Upper gastrointestinal complications among patients with chronic diseases were very common. Fortunately, the symptoms were mild in most cases. The risk increased with age and a using higher number of medications. It is important to review patients’ medications and avoid overuse of them, in addition to use gastro-protective agents when needed.

## Data Availability

The datasets used and/or analyzed during the current study are available from the corresponding author on reasonable request.
